# Validation of the most cost-effective nudge to promote workers’ regular self-weighing: a cluster randomized controlled trial

**DOI:** 10.1038/s41598-022-18916-z

**Published:** 2022-09-15

**Authors:** Masaki Takebayashi, Nobuo Yoshiike, Tatsuya Koyama, Makiko Toriyabe, Hiromi Nakamura, Kurenai Takebayashi

**Affiliations:** 1 Aomori University of Health and Welfare, Hamadate-Maze 58-1, Aomori, 030-8508 Japan; 2 Aomori University, Aomori, Japan; 3 Aomori Prefectural Government, Aomori, Japan

**Keywords:** Health care, Risk factors

## Abstract

Regular self-weighing is useful in obesity prevention. The impact of nudge-based occupational self-weighing programs in the cluster randomized controlled trial was examined. The primary outcome was regular self-weighing after 6 months, which we used to compute cost-effectiveness. Participants were Japanese local government employees who underwent 1 h workshops after being assigned to one of the three nudge groups. Each group was designed according to the nudges’ Easy, Attractive, Social, Timely framework: quiz group (n = 26, attractive-type nudges), implementation intentions group (n = 25, social-type nudges), and growth mindset group (n = 25, timely type nudges). A reference group (n = 36, no nudges) was also formed. After 6 months, all three interventions were effective for regular self-weighing, with the growth mindset intervention (60.0%) being significantly more effective. The cost-effectiveness of the growth mindset group was 1.7 times and 1.3 times higher than that of the quiz group and the implementation intentions group, respectively. Findings from our study are expected to facilitate the use of nudges for health practitioners and employers, which in turn may promote obesity prevention.

## Introduction

Obesity is an important public health issue. The World Health Organization claims that obesity is largely preventable^1^. In Japan, obesity is defined^2^ when an individual has a body mass index (BMI) ≥ 25 kg/m^2^, and its prevalence has been increasing among the working generation lately^3^. In 2013, the percentage of men in their 20 s–60 s with a BMI ≥ 25 kg/m^2^ was 29.0%, and in 2019, it increased to 35.1%^3^. This is higher than the target percentage of 28% in 2022, as stated in the Japanese government's health promotion policy, “Health Japan 21 (second term)”^3^, which emphasizes the aspect of prevention. For example, one of the indicators is the “Increase in percentage of individuals maintaining ideal body weight” as, to promote obesity prevention, it is essential to control weight, even for people without obesity. Although many weight-loss programs have been developed for obese workers^4,5^, the prevalence of obesity continues to increase. Consequently, obesity prevention programs that include non-obese workers who may not have a strong motivation for obesity prevention are required.

Self-weighing is a simple self-monitoring action that can help in obesity prevention. The National Institutes of Health in the U.S. recommends self-monitoring as a crucial component of long-term weight maintenance^6^. Previous studies have shown that frequent self-weighing may be beneficial for weight control and the prevention of weight gain^7^. Daily or weekly self-weighing (hereafter referred to as regular self-weighing) is reported to be correlated with successful weight loss and maintenance^8^. Self-weighing requires very little time and may potentially match the needs of busy workers. However, only 52.4% of the Japanese working generation has been reported to perform regular self-weighing^9^.

Regular self-weighing is a typical intertemporal choice (although the costs occur right now, and the effects appear in the future). Nudges can be useful in making rational intertemporal choices. A nudge is “any aspect of the choice architecture that alters people's behavior in a predictable way without forbidding any options or significantly changing their economic incentives”^10^. One of the nudges’ frameworks is the EAST, which comprises the following elements: “Easy” (e.g., simplifying messages), “Attractive” (e.g., attracting attention), “Social” (e.g., making a commitment), and “Timely” (e.g., prompting at the best timing)^11^. The Japanese government encourages the use of nudges in the workplace for health promotion^12^, but some companies still do not incorporate them into obesity prevention. One of the obstacles to the widespread adoption of nudges could be the difficulty in choosing the highest-priority EAST framework items. Moreover, cramming four nudge elements into one intervention can cause an information overload, which does not match the “Easy” element. Nudges are cost-effective methods^13^, and showing which nudge is the most cost-effective will facilitate health practitioners’ and employers’ decision-making.

This study compared three types of nudge-based programs, the attractive-, social-, and timely type nudges, based on the hypothesis that different nudges will cause changes in self-weighing behavior. We calculated the cost-effectiveness of each intervention according to our previous study^14^. This study aimed to validate the most cost-effective nudge to promote workers’ regular self-weighing after 6 months.

## Methods

### Research design

A cluster randomized controlled trial was conducted with an intention to treat among three nudge groups. A reference group was additionally created to compare the effects between nudges and information provision.

### Setting and participants

Participants were employees at the Aomori Prefectural Government Office located in the Shimokita region who had applied for a self-weighing promotion workshop hosted by the public health center. The program was initiated in September 2017, and a final survey was conducted in March 2018. The total duration of this study (6 months) was decided by referring to behavioral maintenance spans, which were defined in the transtheoretical model^15^. Recruitment was conducted 2 weeks before the first workshop using bulletins. Employees who volunteered were enrolled regardless of sex, age, or BMI, provided that they met the criterion of self-weighing less than once per week.

### Allocation

The employees were working in a section, each of which was positionally remote from another section in their organization. Thus, clusters of participants were formed as section units. Each cluster was numbered and randomly assigned to one of the three nudge groups (quiz, implementation intentions, and growth mindset) using a random number generator.

Clustering and allocation were performed by the first author in accordance with the CONSORT 2010 statement^16^. Although the clustering and allocation procedures were not revealed to the participants until the completion of allocation, they were informed that the study was an RCT.

### Intervention

The programs were designed according to the nudge framework of EAST. All interventions included easy-type nudges. Each nudge group had an assigned type of nudge as its form of intervention. The quiz group mainly used attractive-type nudges, the implementation intentions group used social-type nudges, and the growth mindset group used timely-type nudges.

- Overview of the workshop
  1-hour assembly-type workshops were held at the local government meeting room on separate days in September 2017. Each workshop comprised a general session, an educational session, and a group work session, whose outline is described in the process evaluation paper^14^.
  In the general session, the moderators, who are health center staff, explained the purpose and ethics of the workshop. They also conducted an educational session on the necessity of obesity prevention, effects of weight recording, and importance of a healthy diet and physical activity. After that, a group work session was held wherein the different interventions were applied. However, for the growth mindset group, the order was changed; first, the general session, second, a group work session, and third, the educational session.
- Group work sessions (30 min)
  Group work sessions were conducted in teams of 4–5 participants randomly selected from the roster of each nudge group.
- Quiz group [attractive-type nudge]
  A quiz competition was organized among the teams at the workshop, wherein the team members worked together to answer questions on the costs of obesity (e.g., Question: “In 40 year-old women, how much higher are the medical costs for obese people than for people of appropriate weight”? Answer: “approximately 20%”^17^). Each team discussed their answers for a minute and presented them in turn. The moderator then announced the answer and score. Each team competed for the highest total score from four questions.
  In this group, the following nudges were designed.
  1. An appeal to loss aversion^18^
  2. Addition of game elements to reduce stress induction
- Implementation intentions group [social-type nudge]
  The participants were instructed to declare to their teams the time and place of self-weighing and how they would reward themselves after self-weighing continuously for 1 month. They were also asked to listen attentively to the other member’s declarations, nod, and make sympathetic comments. After that, two moderators demonstrated it as follows: one declared, “I will weigh myself after drying my hair in the washroom. If I continue self-weighing, then I will open an expensive bottle of champagne”. The other said, “Fantastic! I believe you can do it.”
  In this group, the following nudges were designed such that:
  1. Declaration of implementation intentions^19^ leads to commitment, and the positive feedback from others helps to reinforce the commitment. A systematic review reported the positive effect of commitment on weight loss^20^.
  2. By listening to the declarations of others, a peer effect will be fostered.
- Growth mindset group [timely type nudge]
  Participants were instructed to present their experience of achieving success after making an effort. Any kind of experience could be presented, even those that had nothing to do with self-weighing. The moderators explained the instructions as with the implementation intentions group. After that, two moderators demonstrated it as follows: one said, “When I was a high school student, I was short. I practiced a lot and won the regular player in my club.” The other said, “It must be a fantastic experience.”
  The program was designed with the nudges:
  1. Reminding individuals of their achievements and receiving positive feedback could aid in creating a growth mindset among participants (such as “No matter the kind of a person I am, I can always change substantially”)^21^.
  2. Changing the order of the session such that the priming effect could work; the participants would accept the necessity of self-weighing positively when they have a growth mindset.

### Survey of the three nudge groups

The following items were chosen to create a survey in the form of a self-administered questionnaire:

1. *Basic characteristics* Participant’s sex, age, weight, height, BMI, and smoking habits were obtained.
2. *Outcomes* The primary outcome was the number of subjects who had self-weighing habit after 6 months, which was divided by the estimated cost of each program for the cost-effectiveness analysis. The secondary outcomes were changes in behavioral stage or mindset and weight maintenance. In addition, the following presence factors were determined as they were implicated to have a positive effect on weight management: support from others, recording of regular weight measurements, and use of scales distributed at the workplace^22–24^.

The questionnaires for the three groups were managed with a linked-anonymized list. The surveys were conducted at four time intervals: immediately before the workshop (T0), immediately after the workshop (T1), and 6 months after the workshop (T3). In the T3 survey, the self-weighing habits 1 month after the workshop (T2) were also asked. The T0 and T1 survey questionnaires were distributed and collected onsite by the moderators. The T2 and T3 survey were distributed by each organization. Participants sealed their questionnaires in envelopes and placed them inside a collection bag; each department submitted their collection bags to the moderators.

### Reference group

Because of the nature of the workshop, it was difficult to assign applicants to a control group. Therefore, employees from randomly selected organizations of the prefectural government were assigned to the reference group (eligibility criteria: self-weighing frequency was less than once per week). In September 2017, the members of the reference group received a bulletin that contained the same information presented in the educational session conducted in the workshop. The survey questionnaire was administered to the reference group in March 2018 after reminding the participants of their self-weighing habits and weights in September 2017. They were then asked to report their current self-weighing habits and weights.

### Ethical considerations

The management of all the organizations participating in the study agreed to their employees’ participation. The applicants provided written informed consent after receiving a written explanation about the free nature of participation and confidentiality terms. All questionnaires were implemented in an anonymous format. For the three nudge groups, participants received written and verbal notification at the workshop that their responses to the survey would be interpreted as consent. This study was approved by the research ethics committee of the Aomori University of Health and Welfare (Approval no. 1720) and was registered with the UMIN Clinical Trials Registry (UMIN000028143: 08/07/2017). All methods were performed in accordance with the Declaration of Helsinki and adhered to Good Clinical Practice guidelines. All members were given the opportunity to attend a regular weight measurement seminar in 2019, based on this study’s results.

### Distribution of weighing scales to the offices

In the preliminary survey, we found that some employees did not own weighing scales. Therefore, scales were distributed to all the offices, including those in the reference group, to create an environment that facilitates self-weighing.

### Statistical analyses

Assuming a power of 80%, an α error of 5%, and an effect size of 30%, a sample size of 108 participants (36 in each group) was computed based on the primary outcome. Missing values were excluded from the analysis. All analyses were conducted at the individual level. Continuous variables were analyzed using analysis of variance, and categorical data were analyzed using the χ^2^ test, Fisher’s exact test, or Kruskal–Wallis test. SPSS version 24 (IBM, Tokyo) was used for data analysis, and *p* < 0.05 (two-tailed test) was considered to be statistically significant. Residual analysis or the Bonferroni method was used for testing multiple groups.

## Results

**Figure 1:**
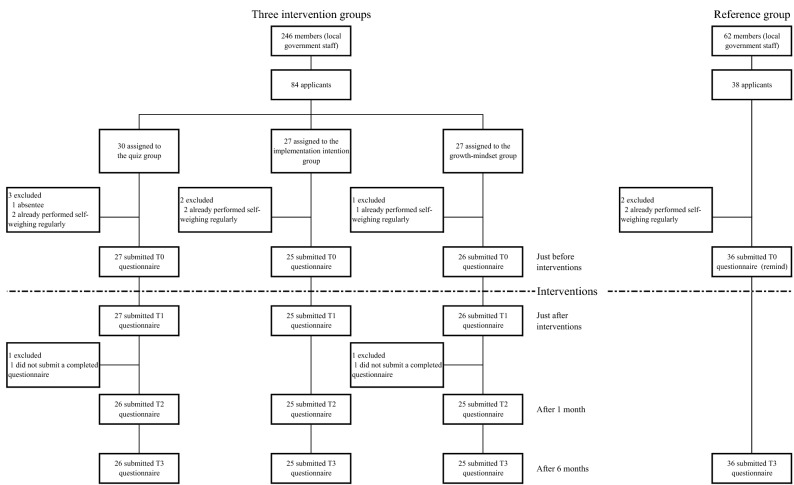
Trial profile of the three nudge groups and reference group.

In the three nudge groups, 84 employees from a total of 246 applied to the self-weighing promotion workshop (Fig. 1). From the preliminary survey, approximately 127 employees did not weigh themselves regularly. In the reference group, the survey was returned by 38 employees. Of these, two did not meet the criteria; thus, 36 were included in the analysis. No adverse events were observed throughout this study.

### Baseline characteristics of the participants

Table 1 displays the baseline characteristics of the participants. The average age ranged from 35.0 to 39.4 years, and the average BMI ranged from 23.0 to 24.3 kg/m^2^. None of the baseline characteristics significantly differed among the groups.

**Table 1:** Baseline characteristics of participants [mean ± SD, n (%)].

	Quiz group (n = 26)	Implementation intentions group (n = 25)	Growth mindset group (n = 25)	Reference group (n = 36)	*p*-value	
					Among four groups	Among three groups (without Reference group)
Age (years)	35.0 ± 13.9	38.6 ± 14.7	39.4 ± 12.9	38.1 ± 13.1	.672	.483
Male sex	20 (76.9)	19 (76.0)	14 (56.0)	27 (75.0)	.286	.189
Weight (kg)	68.0 ± 13.2	68.8 ± 14.2	63.3 ± 8.1	67.0 ± 8.5	.326	.241
BMI (kg/m^2^)	23.6 ± 3.6	24.3 ± 4.2	23.0 ± 2.8	23.0 ± 2.4	.420	.459
BMI distribution					.288	.477
BMI < 18.5	2 (7.7)	1 (4.0)	0 (0)	1 (2.8)		
18.5 ≤ BMI < 25	16 (61.5)	14 (56.0)	20 (83.3)	28 (77.8)		
25 ≤ BMI < 30	6 (23.1)	7 (28.0)	3 (12.5)	7 (19.4)		
BMI ≥ 30	2 (7.7)	3 (12.0)	1 (4.2)	0 (0)		
Smokers^a^	3 (12.0)	4 (16.0)	2 (8.0)	–	–	.759
Weighing scale owners	10 (38.5)	15 (60.0)	13 (52.0)	19 (52.8*)*	.482	.328
Behavior change stage^b^					–	.482
Pre-contemplation	12 (46.2)	11 (44.0)	7 (29.2)	–		
Contemplation	10 (38.5)	7 (28.0)	12 (50.0)	–		
Preparation	4 (15.4)	7 (28.0)	5 (20.8)	–		
Growth mindset person^c^	21 (80.8)	20 (80.0)	21 (84.0)	–	–	.928
Have concerns about future obesity	14 (53.8)	16 (64.0)	16 (64.0)		–	.691
Frequency of regular self-weighing					.699	.703
Once a year	8 (30.8)	4 (16.0)	3 (12.0)	10 (27.8)		
2–3 times a year	3 (11.5)	3 (12.0)	2 (8.0)	1 (2.8)		
4–10 times a year	4 (15.4)	5 (20.0)	7 (28.0)	8 (22.2)		
1–3 times a month	11 (42.3)	13 (52.0)	13 (52.0)	17 (47.2)		

Missing values were excluded from the analysis. Analysis of variance was used for age, weight, and BMI, and the Kruskal–Wallis test was used for the categorical data of appropriate weight distribution, stage of behavioral change, and frequency of regular weight measurement, while χ^2^ test or Fisher’s exact test (items less than five) was used for the other data.

^a^Current habitual cigarette smokers.

^b^Those not interested in regular self-weighing were included in the pre-contemplation stage; those who thought they may try but not right now, in the contemplation stage; and those who wanted to implement regular self-weighing immediately, in the preparation stage.

^c^Those who answered, “Somewhat agree” or “Agree” to the question, “What do you think about the opinion that ‘you can change your lifestyle even now if you want to’?”.

### Comparison among outcomes

Table 2 displays the comparative outcomes. At T3, regular self-weighing, the primary outcome, was observed in 26.9%, 32.0%, and 60.0% of the participants in the quiz, implementation intentions, and growth mindset groups, respectively, while it was observed in 2.8% of those in the reference group (*p* < 0.001). The growth mindset group showed a significantly higher self-weighing habit than the other intervention groups. There were some significant differences in the secondary outcomes. At T3, 49.3% of individuals in the quiz, implementation intentions, and growth mindset groups maintained their weight (48.0%, 41.7%, and 58.3%, respectively), compared with 11.0% in the reference group (*p* < 0.001). The quiz group showed a significantly lower number of individuals acquiring support from others.

**Table 2:** Comparison of outcomes [n (%)].

	Quiz group (n = 26)	Implementation intentions group (n = 25)	Growth mindset group (n = 25)	Reference group (n = 36)	*p*-value^a^	
					Among four groups	Among three groups (without Reference group)
[T0] Individuals self-weighing regularly^b^	0 (0)	0 (0)	0 (0)	0 (0)	–	–
[T1] Individuals whose behavior stage progressed^c^	17 (65.4)	11 (44.0)	18 (72.0)	–	–	.116
[T2] Individuals self-weighing regularly^b^	14 (53.8)	15 (60.0)	18 (72.0)	–	–	.407
[T3] Individuals whose behavior stage progressed^c^	11 (42.3)	13 (56.0)	17 (68.0)	–	–	.179
[T3] Individuals self-weighing regularly^b^	7 (26.9)	8 (32.0)	15 (60.0)	1 (2.8)	< .001*^d^	.034*^e^
[T3] Individuals who maintained their weights^f^	12 (48.0)	10 (41.7)	14 (58.3)	4 (11.1)	< .001*^d^	.528
[T3] Individuals obtaining scales in 6 months	3 (11.5)	3 (12.5)	5 (20.0)	0(0)	.003	.714
[T3] Individuals obtaining supports from others^g^	0 (0)	9 (39.1)	8 (32.0)	–	–	< .001*^h^
[T3] Individuals who recorded their weight^i^	8 (30.8)	5 (20.8)	6 (25.0)	–	–	.715
[T3] Individuals who used scales at office	12 (46.2)	14 (56.0)	14 (58.3)	1 (2.8)	< .001*^d^	.741

Analysis excluding missing values, analysis of variance for continuous value data, χ^2^ test, or Fisher’s exact test (items less than 5).

[T0] immediately before the workshop, [T1] immediately after the workshop, [T2] 1 month after the workshop, [T3] 6 months after the workshop.

^a^(*) indicates a significant difference in the multiple group test (1.96 > p or − 1.96 < p in the residual analysis for the χ^2^ test and p < .05/6 = .008 in the Bonferroni method test for Fisher’s exact test).

^b^Those who regularly weigh themselves at least once a week.

^c^Those who progressed to the stages of change (including those who started regular self-weighing) compared with T0.

^d^The reference group was significantly low.

^e^The growth mindset group was significantly high in the three groups.

^f^Those who did not gain weight for 6 months from T0.

^g^Those who answered, “Yes” or “Yes, a little” to the question, “During the past 6 months, did you get support from the people around you regarding self-weighing?”.

^h^The implementation intentions group was significantly higher than the quiz group.

^i^Those who answered “Almost continuously” or “Sometimes” to the question “During the past 6 months, did you keep a record of your regular weight measurements?”.

In a previous study, the total implementation costs, including labor costs, were $2009, $1755, and $2518 for the quiz group, implementation intentions group, and growth mindset group, respectively^14^; therefore, the cost per person for regular self-weighing at 6 months was $287 (= $2009/7), $219 (= $1755/8), and $168 (= $2518/15), respectively. This means that the cost-effectiveness of the growth mindset intervention was 1.7 times and 1.3 times higher than that of the quiz and implementation intentions interventions, respectively.

## Discussion

At T3, the three nudge groups had significantly higher rates of self-weighing than the reference group, which indicates that nudges were effective. Further, we considered which nudge was the most cost-effective among the three groups.

In this population, it appears that the most cost-effective program was the growth mindset group. One variable potentially explaining the positive result might be the support received by the workers from others; eight people received support, and seven continued self-weighing. Several studies showed that support might influence weight management^22,25^. However, this study could not clarify why the implementations intention group was less effective despite receiving more support from others. The growth mindset group's intervention might promote participants to think: “I can do self-weighing if I get support from others.” The effects could be explained by the synergy of priming and a growth mindset. Priming may change people’s subsequent behavior if they are first exposed to certain information^26^. People primed for stereotypes tend to behave as stereotyped^27^. In the initial group work, the priming was designed to break stereotypes such that the participants have an enhanced growth mindset. This could be a good opportunity to make the participants more receptive to the educational session and to boost self-weighing. The order of sessions is important; the positive priming might not have worked if the educational session had been held before the group work. This implies that interventions should be designed from the viewpoint of optimal timing.

The quiz group had the highest cost per person for regular weight measurement; hence, this might not be the most viable program. Moreover, the quiz group alone had no support from others. The quiz program was not interactive, in contrast with the other two intervention programs, which involved sharing positive comments after declarations or presentations by the participants. Interactive programs may be important in obtaining support from others.

The implementation intentions intervention was reported to be cost-effective in the occupational health activity promotion^13^ but had less impact than the growth mindset program in this setting.

There are some limitations to this study. First, the analyzed set of nudge groups was smaller than the estimated sample size. Therefore, the hypothesized differences between the groups might not have been detected. Second, assessments were based on the self-reported recollection of participants who weighed themselves using household scales; thus, the risk of response/recall bias might not be low. To minimize this bias, participants’ weights should be recorded objectively and uniformly in the study, and their data must be documented. However, the recording could be a bottleneck for many participants and was therefore not implemented. Third, the effect of seasonal bias may have impacted weight maintenance among the participants. For example, seasonal variations of metabolic syndrome prevalence have been reported in Japan^28^. To overcome this limitation, a 1 year follow-up study would be required. Fourth, we did not enquire whether the participants had any obesity-related diseases (e.g., hypertension, dyslipidemia, and diabetes). Even if some participants might have had such diseases, we assumed that since they were randomly assigned to each group, there would be no major problems. As the employees’ disease status information is generally securely administrated in the human resource section, it may not be appropriate to obtain such information at the workshop held by an external body. In fact, many workers hesitated to provide information on the matter in the preliminary survey. Fifth, the participants who applied for the workshop might have had a strong motivation for self-weighing; therefore, because of the selection bias, the participants are probably not representative of the general working population in Japan. Sixth, we could not create a control group. The reference group was provided information on the importance of self-weighing, and we could not estimate the effect without interventions. Further studies to overcome these limitations are warranted.

This study has two strengths. First, it involved a comparison with the reference group. Many studies of nudges in public health lifestyle interventions do not have a control group^29^. Using a reference group can be useful when creating a control group is difficult. Owing to the reference group in this study, we found that nudge-based workshops were more effective than printed materials. Second, the results were analyzed from the viewpoint of cost-effectiveness. This will help practitioners and employers who need simple and low-cost methods to enable healthy choices among their employees^30^. This study’s target behavior and interventions were simple: “self-weighing” and a “1 h workshop,” respectively. Demonstrating the cost-effectiveness of simple behaviors and interventions is expected to facilitate the use of nudges for health practitioners and employers, which in turn may help achieve the obesity prevention goals of the Japanese government^3^. Our findings will provide the basis for further studies regarding obesity prevention.

## Data Availability

The datasets generated during and/or analyzed during the current study are available here: https://1drv.ms/x/s!AvBsmvvF_CGNgw93p-y5JwIgdsFL?e=rM9eQn.
